# Correction: Ultrahigh photocatalytic hydrogen evolution of linear conjugated terpolymers enabled by an ultra-low ratio of the benzothiadiazole monomer

**DOI:** 10.1039/d5sc90121a

**Published:** 2025-06-09

**Authors:** Zheng-Hui Xie, Gang Ye, Hao Gong, Pachaiyappan Murugan, Can Lang, Yi-Fan Dai, Kai Yang, Shi-Yong Liu

**Affiliations:** a School of Chemical Engineering, Guangdong University of Petrochemical Technology Maoming Guangdong 525000 China chelsy@zju.edu.cn; b Jiangxi Provincial Key Laboratory of Functional Crystalline Materials Chemistry, Department of Chemistry and Chemical Engineering, Jiangxi University of Science and Technology Ganzhou 341000 China; c Key Laboratory for the Green Preparation and Application of Functional Materials, Hubei Key Laboratory of Polymer Materials, School of Materials Science and Engineering, Hubei University Youyi Road 368 Wuhan 430062 China; d Center for Global Health Research, Saveetha Medical College and Hospital, Saveetha Institute of Medical and Technical Sciences Kancheepuram District Tamil Nadu India

## Abstract

Correction for ‘Ultrahigh photocatalytic hydrogen evolution of linear conjugated terpolymers enabled by an ultra-low ratio of the benzothiadiazole monomer’ by Zheng-Hui Xie *et al.*, *Chem. Sci.*, 2025, DOI: https://doi.org/10.1039/d5sc01438g.

The authors regret that there are a number of typographical errors in the original version of the article. On the right hand side of page 1, the ref. “24*a*–*c*” should read “24” and the ref. “25*a*–*c*” should read “25”. On the left hand side of page 5 “1.53 eV” should read “1.23 eV”. On page 11, the journal name for ref. 24(*b*) should read “*Chem. Eur. J.*”.

The Royal Society of Chemistry apologises for these errors and any consequent inconvenience to authors and readers.

